# (2,9-Dimethyl-1,10-phenanthroline-κ^2^ *N*,*N*′)bis­(2-meth­oxy­benzoato-κ^2^ *O* ^1^,*O* ^1′^)cadmium

**DOI:** 10.1107/S1600536812010835

**Published:** 2012-03-17

**Authors:** Heng Zhang, Pei-Zheng Zhao

**Affiliations:** a College of Chemistry and Environmental Science, Henan Normal University, Xinxiang 453007, People’s Republic of China

## Abstract

In the title compound, [Cd(C_8_H_7_O_3_)_2_(C_14_H_12_N_2_)], the Cd^II^ ion is coordinated by two N atoms from a 2,9-dimethyl-1,10-phenanthroline (dmphen) ligand and four O atoms from two 2-meth­oxy­benzoate anions in a distorted octa­hedral environment. Two O atoms of one bidentate 2-meth­oxy­benzoate ligand are each disordered over two positions, with site-occupancy factors of 0.579 (4) and 0.421 (4). In the crystal, mol­ecules are linked by C—H⋯O hydrogen bonds, forming a two-dimensional network lieing parallel to the *bc* plane. The crystal packing is further stablized by π–π stacking inter­actions between the dmphen rings of neighboring mol­ecules, with distances between their parallel dmphen ring planes of 3.517 (3) and 3.610 (3) Å.

## Related literature

For features of transition metal complexes with 1,10-phenanthroline and their derivatives, see: Dhar *et al.* (2003 ▶); Mizuno *et al.* (2002 ▶); Wall *et al.* (1999 ▶). For related structures, see: Harvey *et al.* (2000 ▶); Ding *et al.* (2005 ▶); Cui & Zhang (2011 ▶)
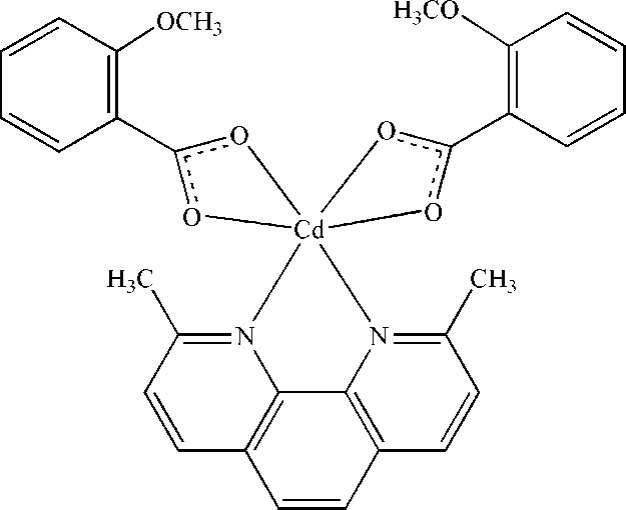


## Experimental

### 

#### Crystal data

[Cd(C_8_H_7_O_3_)_2_(C_14_H_12_N_2_)]
*M* *_r_* = 622.93
Monoclinic, 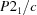

*a* = 16.9045 (12) Å
*b* = 8.0547 (6) Å
*c* = 19.3625 (14) Å
β = 101.877 (1)°
*V* = 2580.0 (3) Å^3^
*Z* = 4
Mo *K*α radiation
μ = 0.90 mm^−1^
*T* = 296 K
0.48 × 0.26 × 0.17 mm

#### Data collection

Bruker SMART CCD area-detector diffractometer
Absorption correction: multi-scan (*SADABS*; Bruker, 2004 ▶) *T*_min_ = 0.673, *T*_max_ = 0.863
18898 measured reflections
4803 independent reflections
4307 reflections with *I* > 2σ(*I*)
*R*_int_ = 0.017

#### Refinement

*R*[*F* ^2^ > 2σ(*F* ^2^)] = 0.023
*wR*(*F* ^2^) = 0.060
*S* = 1.02
4803 reflections
363 parameters
44 restraints
H-atom parameters constrained
Δρ_max_ = 0.59 e Å^−3^
Δρ_min_ = −0.47 e Å^−3^

### 

Data collection: *SMART* (Bruker, 2004 ▶); cell refinement: *SAINT* (Bruker, 2004 ▶); data reduction: *SAINT*; program(s) used to solve structure: *SHELXS97* (Sheldrick, 2008 ▶); program(s) used to refine structure: *SHELXL97* (Sheldrick, 2008 ▶); molecular graphics: *SHELXTL* (Sheldrick, 2008 ▶); software used to prepare material for publication: *publCIF* (Westrip, 2010 ▶).

## Supplementary Material

Crystal structure: contains datablock(s) I, global. DOI: 10.1107/S1600536812010835/bg2438sup1.cif

Structure factors: contains datablock(s) I. DOI: 10.1107/S1600536812010835/bg2438Isup2.hkl

Additional supplementary materials:  crystallographic information; 3D view; checkCIF report

## Figures and Tables

**Table 1:** Hydrogen-bond geometry (Å, °)

*D*—H⋯*A*	*D*—H	H⋯*A*	*D*⋯*A*	*D*—H⋯*A*
C20—H20⋯O5^i^	0.93	2.46	3.312 (3)	152
C17—H17⋯O6^ii^	0.93	2.60	3.487 (3)	161
C14—H14*A*⋯O4	0.96	2.45	3.322 (3)	150
C6—H6⋯O5^iii^	0.93	2.55	3.206 (3)	128

Symmetry codes: (i) 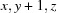; (ii) 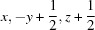; (iii) 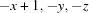.

**Fig. 1.**
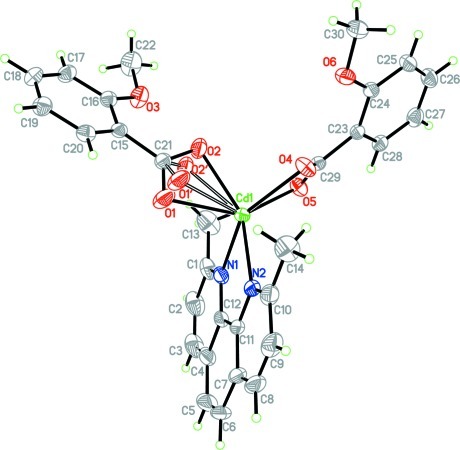
The molecular structure of the title complex(I), with atom labels and 30% probability displacement ellipsoids.

**Fig. 2.**
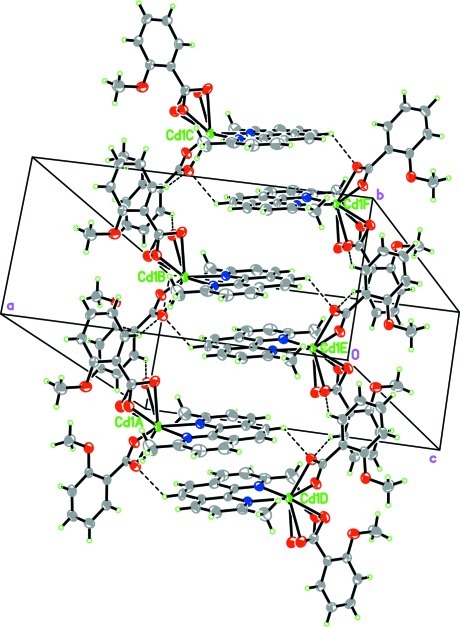
The hydrogen-bonding motifs in the crystal structure of (I). Dashed lines indicate hydrogen bonds and π-π interaction between the dmphen rings of neighboring molecules in the crystal structure of (I).[Symmetry codes:(B)x, y + 1, z; (C)x, y + 2, z; (D)-x + 1, -y, -z; (E)-x + 1, -y + 1, -z; (F)-x + 1, -y + 2, -z + 1/2]

## supplementary crystallographic information

### Comment

The transition metal complexes with 1,10-phenanthroline and their derivatives have attracted much attention because of their peculiar features (Dhar *et al.*, 2003; Mizuno *et al.*, 2002; Wall *et al.*, 1999). Some Cd(II)-phenanthroline complexes have been synthesized and their structures were determined (Harvey *et al.*, 2000; Ding *et al.*, 2005; Cui *et al.*, 2011). Recently, we obtained the title Cadmium(II) complex which contains two different kinds of chelating ligands, by reaction of 2,9-dimethyl-1,10-phenanthroline, 2-methoxy-benzoate and cadmium acetate in an ethanol/water mixture. The structure of the title compound, Cd(C_14_H_12_N_2_)(C_8_H_7_O_3_)_2_,(I), is presented below.

The Cd^II^ ion is coordinated by a bidentate 2,9-dimethyl-1,10-phenanthroline and two bidentate 2-methoxy-benzoate ligands (Fig.1). The CdO_4_N_2_ unit forms a distorted octahedron geometry. Two O atoms of one bidentate 2-methoxy-benzoate are disordered over two positions, with site occupancy factors of *ca* 0.579 (4) and 0.421 (4).

In the crystal structure, molecules are linked into a broad one-dimensional framework by C—H···O hydrogen bonds and π-π stacking interactions between the dmphen rings of neighboring molecules, where vicinal aromatic groups present a face-to-face separations of 3.517 (3) and 3.610 (3) Å. (Fig. 2).

### Experimental

2,9-dimethyl-1,10-phenanthroline hemihydrate (C_14_H_12_N_2_.0.5H_2_O, 0.1086 g, 0.5 mmol) was dissolved in ethanol (10 ml) and Cd(CH_3_COO)_2_.2H_2_O (0.1333 g, 0.5 mmol) in distilled water (5 ml) were added. This solution was added to a solution of 2-methoxy-benzoic acid (C_8_H_8_O_3_, 0.1522 g, 1 mmol) in ethanol (5 ml). The mixture was stirred at 323 K and then refluxed for 11 h, cooled to room temperature and filtered. Colourless single crystals of (I) appeared over a period of two weeks by slow evaporation of the mixture at room temperature.

### Refinement

Methyl H atoms were placed in calculated positions,with C—H=0.96 Å, and refined with free torsion angles to fit the electron density; *U*_iso_(H) = 1.5*U*_eq_(carrier). Other H atoms were placed in calculated positions, with C—H=0.93 Å, and refined in the riding-model approximation with *U*_iso_(H) = 1.2*U*_eq_(C). Two O atoms of one bidentate 2-methoxy-benzoate are disordered over two positions, with site occupancy factors of 0.579 (4) and 0.421 (4). The disordreed moieties were refined with similarity restraints both in distances as in U's.

### Crystal data

**Table:**

[Cd(C_8_H_7_O_3_)_2_(C_14_H_12_N_2_)]	*F*(000) = 1264
*M**_r_* = 622.93	*D*_x_ = 1.604 Mg m^−^^3^
Monoclinic, *P*2_1_/*c*	Mo *K*α radiation, λ = 0.71073 Å
Hall symbol: -P 2ybc	Cell parameters from 9957 reflections
*a* = 16.9045 (12) Å	θ = 2.5–28.2°
*b* = 8.0547 (6) Å	µ = 0.90 mm^−^^1^
*c* = 19.3625 (14) Å	*T* = 296 K
β = 101.877 (1)°	Block, colourless
*V* = 2580.0 (3) Å^3^	0.48 × 0.26 × 0.17 mm
*Z* = 4	

### Data collection

**Table:**

Bruker SMART CCD area-detector diffractometer	4803 independent reflections
Radiation source: fine-focus sealed tube	4307 reflections with *I* > 2σ(*I*)
Graphite monochromator	*R*_int_ = 0.017
phi and ω scans	θ_max_ = 25.5°, θ_min_ = 2.5°
Absorption correction: multi-scan (*SADABS*; Bruker, 2004)	*h* = −20→20
*T*_min_ = 0.673, *T*_max_ = 0.863	*k* = −9→9
18898 measured reflections	*l* = −23→23

### Refinement

**Table:**

Refinement on *F*^2^	Primary atom site location: structure-invariant direct methods
Least-squares matrix: full	Secondary atom site location: difference Fourier map
*R*[*F*^2^ > 2σ(*F*^2^)] = 0.023	Hydrogen site location: inferred from neighbouring sites
*wR*(*F*^2^) = 0.060	H-atom parameters constrained
*S* = 1.02	*w* = 1/[σ^2^(*F*_o_^2^) + (0.0297*P*)^2^ + 1.5264*P*] where *P* = (*F*_o_^2^ + 2*F*_c_^2^)/3
4803 reflections	(Δ/σ)_max_ = 0.001
363 parameters	Δρ_max_ = 0.59 e Å^−^^3^
44 restraints	Δρ_min_ = −0.47 e Å^−^^3^

### Special details

**Table:**

Geometry. All e.s.d.'s (except the e.s.d. in the dihedral angle between two l.s. planes)are estimated using the full covariance matrix. The cell e.s.d.'s are takeninto account individually in the estimation of e.s.d.'s in distances, anglesand torsion angles; correlations between e.s.d.'s in cell parameters are onlyused when they are defined by crystal symmetry. An approximate (isotropic)treatment of cell e.s.d.'s is used for estimating e.s.d.'s involving l.s. planes.
Refinement. Refinement of *F*^2^ against ALL reflections. The weighted *R*-factor *wR* and goodness of fit *S* are based on *F*^2^, conventional *R*-factors *R* are based on *F*, with *F* set to zero for negative *F*^2^. The threshold expression of *F*^2^ > σ(*F*^2^) is used only for calculating *R*-factors(gt) *etc*. and is not relevant to the choice of reflections for refinement. *R*-factors based on *F*^2^ are statistically about twice as large as those based on *F*, and *R*- factors based on ALL data will be even larger.

### Fractional atomic coordinates and isotropic or equivalent isotropic displacement parameters (Å^2^)

**Table:**

	*x*	*y*	*z*	*U*_iso_*/*U*_eq_	Occ. (<1)
O1	0.30422 (17)	0.4057 (4)	0.12323 (16)	0.0450 (4)	0.579 (4)
O2	0.20884 (17)	0.2183 (4)	0.12535 (17)	0.0494 (4)	0.579 (4)
O1'	0.2860 (3)	0.4050 (6)	0.1036 (2)	0.0450 (4)	0.421 (4)
O2'	0.2352 (3)	0.1914 (5)	0.1509 (2)	0.0494 (4)	0.421 (4)
Cd1	0.307440 (8)	0.144637 (19)	0.062350 (7)	0.03648 (5)	
O3	0.15835 (11)	0.2486 (2)	0.25184 (8)	0.0552 (4)	
O4	0.20186 (9)	0.05972 (19)	−0.02720 (8)	0.0483 (4)	
O5	0.25548 (9)	−0.12268 (18)	0.05286 (8)	0.0460 (4)	
O6	0.04132 (8)	−0.00151 (19)	−0.07279 (8)	0.0473 (4)	
N1	0.43234 (10)	0.0689 (2)	0.13255 (9)	0.0425 (4)	
N2	0.40861 (10)	0.2261 (2)	0.00289 (9)	0.0404 (4)	
C1	0.44175 (16)	−0.0095 (3)	0.19443 (12)	0.0563 (6)	
C2	0.51880 (18)	−0.0427 (4)	0.23466 (15)	0.0736 (9)	
H2	0.5244	−0.0980	0.2775	0.088*	
C3	0.58528 (18)	0.0059 (4)	0.21110 (17)	0.0778 (9)	
H3	0.6364	−0.0158	0.2381	0.093*	
C4	0.57759 (14)	0.0884 (3)	0.14638 (16)	0.0625 (7)	
C5	0.64459 (16)	0.1445 (4)	0.1188 (2)	0.0816 (10)	
H5	0.6968	0.1268	0.1445	0.098*	
C6	0.63385 (15)	0.2224 (4)	0.0567 (2)	0.0784 (9)	
H6	0.6789	0.2588	0.0404	0.094*	
C7	0.55435 (14)	0.2517 (3)	0.01409 (15)	0.0587 (6)	
C8	0.54051 (17)	0.3281 (3)	−0.05163 (17)	0.0725 (8)	
H8	0.5840	0.3638	−0.0704	0.087*	
C9	0.4639 (2)	0.3511 (3)	−0.08871 (15)	0.0700 (8)	
H9	0.4551	0.4006	−0.1330	0.084*	
C10	0.39695 (15)	0.2994 (3)	−0.05982 (12)	0.0520 (6)	
C11	0.48584 (12)	0.2002 (3)	0.03993 (12)	0.0443 (5)	
C12	0.49803 (13)	0.1175 (3)	0.10742 (13)	0.0448 (5)	
C13	0.36708 (19)	−0.0598 (4)	0.21911 (14)	0.0796 (9)	
H13A	0.3348	0.0366	0.2227	0.119*	
H13B	0.3819	−0.1120	0.2645	0.119*	
H13C	0.3366	−0.1364	0.1860	0.119*	
C14	0.31198 (18)	0.3251 (4)	−0.09904 (14)	0.0695 (8)	
H14A	0.2806	0.2278	−0.0946	0.104*	
H14B	0.3117	0.3450	−0.1480	0.104*	
H14C	0.2891	0.4190	−0.0797	0.104*	
C15	0.20540 (11)	0.4688 (3)	0.18954 (10)	0.0353 (4)	
C16	0.16481 (12)	0.4150 (3)	0.24222 (10)	0.0387 (5)	
C17	0.13494 (13)	0.5310 (3)	0.28312 (12)	0.0479 (5)	
H17	0.1091	0.4955	0.3185	0.057*	
C18	0.14315 (14)	0.6992 (3)	0.27181 (13)	0.0516 (6)	
H18	0.1228	0.7758	0.2996	0.062*	
C19	0.18119 (13)	0.7540 (3)	0.21972 (12)	0.0482 (5)	
H19	0.1862	0.8670	0.2118	0.058*	
C20	0.21190 (13)	0.6384 (3)	0.17919 (11)	0.0416 (5)	
H20	0.2376	0.6755	0.1440	0.050*	
C21	0.24280 (12)	0.3527 (3)	0.14442 (11)	0.0405 (4)	
C22	0.1085 (2)	0.1952 (4)	0.29900 (16)	0.0758 (8)	
H22A	0.1299	0.2374	0.3454	0.114*	
H22B	0.1076	0.0761	0.3004	0.114*	
H22C	0.0545	0.2361	0.2828	0.114*	
C23	0.13813 (11)	−0.2080 (3)	−0.02853 (10)	0.0351 (4)	
C24	0.05986 (12)	−0.1656 (3)	−0.06558 (10)	0.0384 (5)	
C25	0.00480 (13)	−0.2911 (3)	−0.09126 (12)	0.0495 (6)	
H25	−0.0467	−0.2638	−0.1161	0.059*	
C26	0.02634 (15)	−0.4555 (3)	−0.07995 (13)	0.0562 (6)	
H26	−0.0107	−0.5380	−0.0978	0.067*	
C27	0.10208 (15)	−0.4998 (3)	−0.04248 (12)	0.0519 (6)	
H27	0.1160	−0.6109	−0.0346	0.062*	
C28	0.15693 (13)	−0.3753 (3)	−0.01683 (11)	0.0422 (5)	
H28	0.2077	−0.4043	0.0089	0.051*	
C29	0.20123 (11)	−0.0812 (3)	0.00066 (10)	0.0345 (4)	
C30	−0.03947 (14)	0.0418 (4)	−0.10615 (14)	0.0611 (7)	
H30A	−0.0494	0.0086	−0.1548	0.092*	
H30B	−0.0465	0.1597	−0.1033	0.092*	
H30C	−0.0768	−0.0138	−0.0828	0.092*	

### Atomic displacement parameters (Å^2^)

**Table:**

	*U*^11^	*U*^22^	*U*^33^	*U*^12^	*U*^13^	*U*^23^
O1	0.0453 (7)	0.0492 (6)	0.0427 (9)	0.0018 (6)	0.0146 (7)	−0.0052 (6)
O2	0.0472 (8)	0.0521 (7)	0.0521 (9)	−0.0004 (6)	0.0172 (7)	−0.0110 (7)
O1'	0.0453 (7)	0.0492 (6)	0.0427 (9)	0.0018 (6)	0.0146 (7)	−0.0052 (6)
O2'	0.0472 (8)	0.0521 (7)	0.0521 (9)	−0.0004 (6)	0.0172 (7)	−0.0110 (7)
Cd1	0.02953 (8)	0.04385 (9)	0.03711 (8)	−0.00541 (6)	0.00932 (6)	−0.00570 (6)
O3	0.0769 (11)	0.0434 (9)	0.0519 (9)	−0.0021 (8)	0.0286 (8)	0.0021 (7)
O4	0.0421 (8)	0.0463 (9)	0.0512 (9)	−0.0114 (7)	−0.0027 (7)	0.0072 (7)
O5	0.0435 (8)	0.0485 (9)	0.0412 (8)	−0.0067 (7)	−0.0027 (7)	0.0022 (6)
O6	0.0338 (7)	0.0507 (9)	0.0547 (9)	0.0001 (7)	0.0029 (6)	0.0011 (7)
N1	0.0392 (9)	0.0404 (9)	0.0450 (10)	−0.0038 (8)	0.0015 (8)	−0.0075 (8)
N2	0.0408 (9)	0.0401 (9)	0.0438 (9)	−0.0072 (8)	0.0170 (8)	−0.0088 (8)
C1	0.0659 (15)	0.0497 (14)	0.0444 (13)	−0.0037 (12)	−0.0096 (11)	−0.0048 (11)
C2	0.0766 (19)	0.0648 (17)	0.0639 (17)	0.0070 (15)	−0.0215 (15)	−0.0022 (14)
C3	0.0603 (16)	0.0705 (18)	0.084 (2)	0.0167 (15)	−0.0292 (15)	−0.0167 (16)
C4	0.0367 (12)	0.0559 (14)	0.0887 (19)	0.0029 (11)	−0.0017 (12)	−0.0290 (14)
C5	0.0339 (13)	0.077 (2)	0.130 (3)	0.0012 (13)	0.0076 (16)	−0.0381 (19)
C6	0.0363 (12)	0.0722 (18)	0.137 (3)	−0.0151 (12)	0.0415 (15)	−0.0456 (19)
C7	0.0500 (12)	0.0493 (13)	0.0879 (17)	−0.0141 (11)	0.0403 (12)	−0.0275 (13)
C8	0.0705 (16)	0.0641 (17)	0.101 (2)	−0.0256 (13)	0.0593 (16)	−0.0288 (15)
C9	0.099 (2)	0.0605 (16)	0.0643 (16)	−0.0215 (15)	0.0495 (16)	−0.0069 (13)
C10	0.0627 (14)	0.0468 (12)	0.0520 (13)	−0.0112 (11)	0.0243 (11)	−0.0067 (11)
C11	0.0339 (10)	0.0379 (11)	0.0653 (14)	−0.0075 (9)	0.0198 (10)	−0.0226 (10)
C12	0.0342 (10)	0.0397 (11)	0.0579 (13)	−0.0007 (9)	0.0039 (10)	−0.0179 (10)
C13	0.089 (2)	0.096 (2)	0.0483 (14)	−0.0276 (18)	0.0030 (14)	0.0169 (15)
C14	0.0814 (19)	0.0728 (18)	0.0538 (15)	−0.0059 (15)	0.0128 (14)	0.0141 (13)
C15	0.0286 (9)	0.0458 (11)	0.0301 (9)	0.0018 (8)	0.0026 (7)	−0.0014 (8)
C16	0.0364 (10)	0.0446 (11)	0.0347 (10)	0.0004 (9)	0.0065 (8)	0.0014 (9)
C17	0.0482 (12)	0.0548 (14)	0.0460 (12)	0.0004 (10)	0.0220 (10)	−0.0021 (10)
C18	0.0496 (12)	0.0521 (13)	0.0566 (13)	0.0092 (11)	0.0192 (11)	−0.0078 (11)
C19	0.0484 (12)	0.0401 (12)	0.0558 (13)	0.0055 (10)	0.0103 (10)	0.0040 (10)
C20	0.0373 (11)	0.0501 (12)	0.0376 (11)	−0.0003 (9)	0.0080 (9)	0.0049 (9)
C21	0.0389 (7)	0.0452 (7)	0.0374 (7)	0.0038 (5)	0.0080 (5)	−0.0022 (5)
C22	0.105 (2)	0.0601 (16)	0.0738 (18)	−0.0175 (16)	0.0444 (17)	0.0048 (14)
C23	0.0340 (9)	0.0429 (11)	0.0308 (9)	−0.0057 (9)	0.0121 (8)	−0.0045 (8)
C24	0.0349 (10)	0.0487 (12)	0.0339 (10)	−0.0069 (9)	0.0123 (8)	−0.0047 (9)
C25	0.0380 (11)	0.0634 (15)	0.0467 (12)	−0.0119 (11)	0.0080 (9)	−0.0099 (11)
C26	0.0549 (13)	0.0560 (15)	0.0600 (14)	−0.0227 (11)	0.0170 (11)	−0.0167 (12)
C27	0.0642 (14)	0.0415 (12)	0.0551 (13)	−0.0086 (11)	0.0242 (11)	−0.0073 (10)
C28	0.0433 (11)	0.0467 (12)	0.0395 (11)	−0.0021 (9)	0.0151 (9)	−0.0047 (9)
C29	0.0293 (9)	0.0435 (11)	0.0324 (9)	−0.0020 (8)	0.0107 (8)	−0.0041 (8)
C30	0.0420 (12)	0.0708 (17)	0.0654 (16)	0.0091 (12)	−0.0007 (11)	−0.0055 (13)

### Geometric parameters (Å, º)

**Table:**

O1—C21	1.266 (3)	C8—H8	0.9300
O1—Cd1	2.416 (3)	C9—C10	1.424 (4)
O2—C21	1.245 (3)	C9—H9	0.9300
O2—Cd1	2.336 (3)	C10—C14	1.495 (4)
O1'—C21	1.254 (4)	C11—C12	1.443 (3)
O1'—Cd1	2.299 (5)	C13—H13A	0.9600
O2'—C21	1.314 (4)	C13—H13B	0.9600
O2'—Cd1	2.331 (4)	C13—H13C	0.9600
Cd1—O5	2.3184 (15)	C14—H14A	0.9600
Cd1—O4	2.3207 (14)	C14—H14B	0.9600
Cd1—N2	2.3435 (16)	C14—H14C	0.9600
Cd1—N1	2.3444 (17)	C15—C20	1.388 (3)
Cd1—C29	2.660 (2)	C15—C16	1.410 (3)
Cd1—C21	2.691 (2)	C15—C21	1.505 (3)
O3—C16	1.361 (3)	C16—C17	1.385 (3)
O3—C22	1.430 (3)	C17—C18	1.384 (3)
O4—C29	1.258 (3)	C17—H17	0.9300
O5—C29	1.262 (2)	C18—C19	1.376 (3)
O6—C24	1.359 (3)	C18—H18	0.9300
O6—C30	1.429 (3)	C19—C20	1.385 (3)
N1—C1	1.335 (3)	C19—H19	0.9300
N1—C12	1.359 (3)	C20—H20	0.9300
N2—C10	1.328 (3)	C22—H22A	0.9600
N2—C11	1.370 (3)	C22—H22B	0.9600
C1—C2	1.399 (4)	C22—H22C	0.9600
C1—C13	1.495 (4)	C23—C28	1.392 (3)
C2—C3	1.355 (5)	C23—C24	1.411 (3)
C2—H2	0.9300	C23—C29	1.501 (3)
C3—C4	1.401 (4)	C24—C25	1.395 (3)
C3—H3	0.9300	C25—C26	1.378 (4)
C4—C12	1.419 (3)	C25—H25	0.9300
C4—C5	1.421 (4)	C26—C27	1.382 (3)
C5—C6	1.336 (5)	C26—H26	0.9300
C5—H5	0.9300	C27—C28	1.387 (3)
C6—C7	1.444 (4)	C27—H27	0.9300
C6—H6	0.9300	C28—H28	0.9300
C7—C8	1.389 (4)	C30—H30A	0.9600
C7—C11	1.415 (3)	C30—H30B	0.9600
C8—C9	1.358 (4)	C30—H30C	0.9600
			
C21—O1—Cd1	88.12 (18)	C7—C11—C12	118.7 (2)
C21—O2—Cd1	92.34 (17)	N1—C12—C4	121.1 (2)
C21—O1'—Cd1	93.8 (3)	N1—C12—C11	118.83 (18)
C21—O2'—Cd1	90.8 (2)	C4—C12—C11	120.0 (2)
O1'—Cd1—O5	142.22 (10)	C1—C13—H13A	109.5
O1'—Cd1—O4	112.12 (12)	C1—C13—H13B	109.5
O5—Cd1—O4	56.39 (5)	H13A—C13—H13B	109.5
O1'—Cd1—O2'	56.84 (12)	C1—C13—H13C	109.5
O5—Cd1—O2'	87.78 (10)	H13A—C13—H13C	109.5
O4—Cd1—O2'	99.14 (12)	H13B—C13—H13C	109.5
O5—Cd1—O2	88.74 (8)	C10—C14—H14A	109.5
O4—Cd1—O2	86.75 (8)	C10—C14—H14B	109.5
O1'—Cd1—N2	95.65 (10)	H14A—C14—H14B	109.5
O5—Cd1—N2	121.61 (6)	C10—C14—H14C	109.5
O4—Cd1—N2	104.02 (6)	H14A—C14—H14C	109.5
O2'—Cd1—N2	149.50 (10)	H14B—C14—H14C	109.5
O2—Cd1—N2	148.95 (8)	C20—C15—C16	118.13 (19)
O1'—Cd1—N1	102.91 (12)	C20—C15—C21	118.20 (18)
O5—Cd1—N1	94.91 (6)	C16—C15—C21	123.67 (19)
O4—Cd1—N1	144.97 (6)	O3—C16—C17	122.47 (19)
O2'—Cd1—N1	99.26 (12)	O3—C16—C15	117.81 (18)
O2—Cd1—N1	114.69 (9)	C17—C16—C15	119.7 (2)
N2—Cd1—N1	72.33 (6)	C18—C17—C16	120.6 (2)
O5—Cd1—O1	143.18 (8)	C18—C17—H17	119.7
O4—Cd1—O1	121.99 (8)	C16—C17—H17	119.7
O2—Cd1—O1	55.52 (9)	C19—C18—C17	120.5 (2)
N2—Cd1—O1	95.04 (8)	C19—C18—H18	119.8
N1—Cd1—O1	92.96 (8)	C17—C18—H18	119.8
O1'—Cd1—C29	129.77 (11)	C18—C19—C20	119.1 (2)
O2'—Cd1—C29	91.94 (11)	C18—C19—H19	120.5
O2—Cd1—C29	85.42 (8)	C20—C19—H19	120.5
N2—Cd1—C29	117.66 (6)	C19—C20—C15	122.0 (2)
N1—Cd1—C29	121.76 (6)	C19—C20—H20	119.0
O1—Cd1—C29	137.15 (8)	C15—C20—H20	119.0
O1'—Cd1—C21	27.70 (10)	O2—C21—O1'	113.5 (3)
O5—Cd1—C21	115.59 (6)	O2—C21—O1	123.7 (3)
O4—Cd1—C21	106.35 (6)	O1'—C21—O2'	118.2 (3)
O2—Cd1—C21	27.53 (8)	O1—C21—O2'	117.8 (3)
N2—Cd1—C21	122.80 (6)	O2—C21—C15	119.3 (2)
N1—Cd1—C21	104.26 (6)	O1'—C21—C15	121.7 (3)
C29—Cd1—C21	111.87 (6)	O1—C21—C15	116.7 (2)
C16—O3—C22	117.27 (19)	O2'—C21—C15	119.9 (2)
C29—O4—Cd1	91.07 (11)	O2—C21—Cd1	60.13 (15)
C29—O5—Cd1	91.08 (12)	O1'—C21—Cd1	58.5 (2)
C24—O6—C30	117.56 (18)	O1—C21—Cd1	63.82 (17)
C1—N1—C12	120.2 (2)	O2'—C21—Cd1	60.0 (2)
C1—N1—Cd1	124.78 (16)	C15—C21—Cd1	179.11 (14)
C12—N1—Cd1	114.99 (14)	O3—C22—H22A	109.5
C10—N2—C11	119.51 (19)	O3—C22—H22B	109.5
C10—N2—Cd1	126.00 (15)	H22A—C22—H22B	109.5
C11—N2—Cd1	114.41 (14)	O3—C22—H22C	109.5
N1—C1—C2	121.0 (3)	H22A—C22—H22C	109.5
N1—C1—C13	117.6 (2)	H22B—C22—H22C	109.5
C2—C1—C13	121.4 (3)	C28—C23—C24	118.41 (18)
C3—C2—C1	119.9 (3)	C28—C23—C29	118.45 (18)
C3—C2—H2	120.0	C24—C23—C29	123.11 (19)
C1—C2—H2	120.0	O6—C24—C25	123.07 (19)
C2—C3—C4	120.5 (2)	O6—C24—C23	117.39 (17)
C2—C3—H3	119.8	C25—C24—C23	119.5 (2)
C4—C3—H3	119.8	C26—C25—C24	120.3 (2)
C3—C4—C12	117.2 (3)	C26—C25—H25	119.9
C3—C4—C5	123.5 (3)	C24—C25—H25	119.9
C12—C4—C5	119.3 (3)	C25—C26—C27	121.2 (2)
C6—C5—C4	121.1 (3)	C25—C26—H26	119.4
C6—C5—H5	119.5	C27—C26—H26	119.4
C4—C5—H5	119.5	C26—C27—C28	118.7 (2)
C5—C6—C7	122.0 (3)	C26—C27—H27	120.7
C5—C6—H6	119.0	C28—C27—H27	120.7
C7—C6—H6	119.0	C27—C28—C23	121.9 (2)
C8—C7—C11	117.3 (2)	C27—C28—H28	119.1
C8—C7—C6	123.9 (2)	C23—C28—H28	119.1
C11—C7—C6	118.9 (3)	O4—C29—O5	120.88 (18)
C9—C8—C7	120.5 (2)	O4—C29—C23	121.38 (17)
C9—C8—H8	119.8	O5—C29—C23	117.70 (18)
C7—C8—H8	119.8	O4—C29—Cd1	60.71 (10)
C8—C9—C10	120.1 (3)	O5—C29—Cd1	60.61 (10)
C8—C9—H9	120.0	C23—C29—Cd1	175.00 (13)
C10—C9—H9	120.0	O6—C30—H30A	109.5
N2—C10—C9	120.6 (2)	O6—C30—H30B	109.5
N2—C10—C14	118.2 (2)	H30A—C30—H30B	109.5
C9—C10—C14	121.2 (2)	O6—C30—H30C	109.5
N2—C11—C7	122.1 (2)	H30A—C30—H30C	109.5
N2—C11—C12	119.18 (18)	H30B—C30—H30C	109.5

### Hydrogen-bond geometry (Å, º)

**Table:**

*D*—H···*A*	*D*—H	H···*A*	*D*···*A*	*D*—H···*A*
C20—H20···O5^i^	0.93	2.46	3.312 (3)	152
C17—H17···O6^ii^	0.93	2.60	3.487 (3)	161
C14—H14*A*···O4	0.96	2.45	3.322 (3)	150
C6—H6···O5^iii^	0.93	2.55	3.206 (3)	128

Symmetry codes: (i) *x*, *y*+1, *z*; (ii) *x*, −*y*+1/2, *z*+1/2; (iii) −*x*+1, −*y*, −*z*.
